# Growth and weight status in treatment-naïve 12-16 year old adolescents with Alcohol Use Disorders in Cape Town, South Africa

**DOI:** 10.1186/1475-2891-10-87

**Published:** 2011-08-23

**Authors:** Celeste E Naude, Marjanne Senekal, Ria Laubscher, Paul D Carey, George Fein

**Affiliations:** 1 Division of Human Nutrition, Stellenbosch University, South Africa; 2 Division of Human Nutrition, University of Cape Town, South Africa; 3 Biostatistics Unit, Medical Research Council of South Africa, South Africa; 4 Department of Psychiatry, Stellenbosch University, South Africa; 5 Neurobehavioral Research Inc, Hawaii

**Keywords:** adolescent, alcohol, growth, weight, anthropometry, energy intake

## Abstract

**Background:**

Heavy alcohol consumption during adolescence has many known harmful health and social consequences and is strongly associated with numerous health risk behaviours. The consequences of heavy alcohol use during adolescence on nutritional status, specifically growth and weight status are largely unknown at this time.

**Methods:**

Substance use, anthropometric indices of growth and weight, dietary energy intake and physical activity in heavy drinking adolescents (meeting DSM-IV criteria for alcohol use disorders) and matched light/non-drinking control adolescents were assessed.

**Results:**

Lifetime alcohol dose, measured in standard drinks of alcohol, was orders of magnitude higher in adolescents with alcohol use disorders (AUDs) compared to controls. The AUDs group was selected to represent relatively 'pure' AUDs, with minimal other drug use and no psychiatric diagnoses. The growth and weight status of adolescents with AUDs were generally comparable to that of controls, and is in line with the growth and weight status of the South African adolescent population. A greater proportion of overweight/obese females was found in both groups, with this percentage tending to be greater, although not significantly so, in the AUDs group. Adolescent females with AUDs had increased odds of being overweight/obese compared to controls, after adjustment for smoking, physical activity and energy intake.

**Conclusion:**

Anthropometric indices of growth and weight status of participants in the Control and AUD groups were generally comparable. Female adolescents with AUDs may have an increased risk of being overweight/obese compared to adolescent females without AUDs. The presence of an AUD in our adolescent sample was associated with higher energy intake. Longitudinal studies are needed to elucidate the effects of heavy alcohol use on energy balance, growth and weight status in adolescents as they age. Nonetheless, the current study contributes to our understanding of the impacts of heavy alcohol consumption on important aspects of adolescent development.

## Introduction

Heavy alcohol consumption during the crucial developmental period of adolescence is an important public health concern in both in developed [1,2] and developing countries [3,4]. Adolescent alcohol abuse/dependence has many known harmful health and social consequences, such as school failure [5], crime and violence [6], increased risk of adult alcohol dependence/abuse, illicit drug use, social adversity [7] cardiovascular disease [8], and is also strongly associated with a wide range of other health risk behaviours [2]. Examination of the effects of heavy alcohol use during adolescence has expanded in recent years [9], however, the consequences for adolescent nutritional status are largely unknown at this time.

The 2008 South African Youth Risk Behaviour Survey (YRBS) reports that 35% of a national sample of grade 8 to 11 adolescents (*n = 10 270*) reported drinking alcohol on one or more days in the month preceding the survey. Nationally, 29% of adolescents had engaged in past month binge drinking, a significant increase from the rate of 23% in the 2002 YRBS [10]. Significantly more learners in the Western Cape Province (41%) had engaged in binge drinking in the past month when compared to the national average of 29% [4].

Globally, adolescents are recognized as a nutritionally at-risk group [11]. High nutritional demand for growth and development, poor eating behaviour during adolescence [11-13], and a propensity for risk-taking behaviours are all threats to nutritional adequacy [11]. Adolescent eating patterns are typically characterised by high consumption of sweetened beverages, increased intake of energy-dense nutrient-poor foods, and frequently skipping meals, particularly breakfast [13-16]. South African adolescents are no exception [17,18]. Poor nutrition during this life-stage, which is also characterised by the adolescent growth spurt, may be associated with stunting (chronic undernutrition), underweight (chronic negative energy balance), or being overweight or obese (chronic positive energy balance)[11].

Alcohol is energy-dense and energy provided by alcoholic drinks is derived from the alcohol (29 kilojoules per gram) and the carbohydrates (17 kilojoules per gram) they contain, with most drinks containing negligible amounts of other nutrients [19]. Heavy alcohol use may affect total energy intake in a variety of ways. First, if alcohol energy replaces food energy (thus no change in total energy intake) dietary quality is reduced, with poor intake of essential macro- and micronutrients, even though energy needs may be met. This nutrient inadequacy increases the risk for nutrient deficiencies, which may enhance the risk for stunting (low height-for-age) [20]. Second, heavy alcohol use may result in a more significant reduction in dietary intake with energy from alcohol not compensating for the total loss of dietary energy intake. Inadequate energy and nutrient intake could manifest in the adolescent as underweight (low body mass index (BMI)-for-age) or possibly stunting [20]. However, it must be borne in mind, that the greatest risk for stunting remains poor nutrition during the first two years of life [21]. Third, alcohol containing drinks could be ingested in addition to usual food intake, resulting in increased total energy consumption, compounding the risks for weight gain and being overweight/obese (high BMI-for-age) [20].

A recent review examined the outcomes of preload studies of the effects of alcohol on subsequent food intake in adults, and reported that in the short-term, energy ingested as alcohol is additive to energy consumed from other sources, suggesting that alcohol promotes short-term passive over-consumption of energy [22]. According to Yeomans (2010), alcohol is very inefficient at triggering the satiety mechanisms involved in short-term control of food intake. Adult studies have further found alcohol use to be positively associated with BMI or obesity [23-25]. The work by Oesterle and colleagues, namely that chronic heavy drinkers aged 10 to 24 years were nearly four times more likely to be overweight or obese at age 24, suggests that this may be true for adolescents [26]. The positive association between alcohol and tobacco use and unhealthy eating habits such as consuming sweetened carbonated drinks, sweets and snacks, as reported in adolescents [27-30], further compounds these risks. As such, it is reasonable to speculate that dietary changes associated with heavy alcohol use in adolescence may include a higher intake of energy-dense foods, thus contributing to increased total energy intake. Thus, heavy alcohol use during adolescence may promote overweight and obesity via the additive effect of alcohol energy as well as dietary changes favouring energy-dense items, resulting in a persistent positive energy balance.

The 2002 and 2008 YRBSs in South Africa show overnutrition to be a greater problem in this age group than undernutrition, with an increase in the prevalence of overweight and obesity nationally from 2002 to 2008 (21 to 25%), especially in the mixed ancestry population (16.6 to 22.4%). The prevalence of overweight and obesity was consistently greater in females nationally, in the Western Cape Province, and in the mixed ancestry population group [4,10]. This follows a widely recognised global trend in which overweight exceeds underweight in females in more than half of the world's developing countries [31].

Heavy alcohol ingestion can compound the problem of positive energy balance in usual dietary intake, increasing the risk for weight gain. Thus, it is argued that heavy alcohol use during adolescence may increase the risk for overweight and obesity. At this time, studies investigating the association between heavy alcohol use, energy balance, growth and weight status of adolescents are lacking internationally, with no such studies having been conducted in South Africa.

The current study examines anthropometric indices of growth and weight status in treatment-naive, 12 to 16 year old community-dwelling adolescents with alcohol use disorders (AUDs) in comparison to matched light/non-drinking control adolescents, both groups without co-morbid substance use disorders (SUDs) or psychiatric disorders, as part of a larger study exploring the effects of heavy alcohol use on brain structure and function. The inclusion of adolescents without co-morbid externalizing disorders or SUDs, allows us to study the effects of AUDs on growth and weight indices without the confounding effects of other substance abuse or externalising disorder risk factors. It is hypothesised that anthropometric indices of growth and weight status may be different in adolescents with AUDs compared to light/non-drinking control adolescents.

## Methods

### Study Population and Participants

This study examined adolescents with relatively 'pure' AUDs, without concomitant drug use or psychiatric (including externalizing) diagnoses. The sample (*n = 162*) consisted of low socio-economic status English or Afrikaans-speaking adolescents (ages 12 to 16 years) from schools within a 25-km radius of Tygerberg Hospital located in the greater metropolitan area of Cape Town, South Africa. Screening procedures included a structured psychiatric diagnostic interview, a developmental and medical history (from participants and at least one biological parent or legal guardian) and a detailed physical and neurological examination assessing developmental delays. The Schedule for Affective Disorders and Schizophrenia for School Aged Children (six to 18 years) Lifetime Version (K-SADS-PL) [32] was used to screen for psychiatric diagnoses. The Semi-Structured Assessment for the Genetics of Alcohol (SSAGA-II) [33] was used to confirm AUDs diagnosis and to derive detailed substance use histories (alcohol, tobacco and all other drugs).

Participants were assigned to one of two groups: an AUDs group meeting DSM-IV criteria for alcohol dependence or alcohol abuse [34] or a light/non-drinking Control group (lifetime dose of < 100 standard drinks of alcohol or never consumed alcohol). Exclusion criteria for both groups were: mental retardation, lifetime DSM-IV diagnoses other than AUDs (as defined in the KSADS-PL, including major depression, dysthymia, mania, hypomania, cyclothymia, bipolar disorders, schizoaffective disorders, schizophrenia, schizophreniform disorder, brief reactive psychosis, panic disorder, agoraphobia, separation anxiety disorder, avoidant disorder of childhood and adolescence, simple phobia, social phobia, overanxious disorder, generalized anxiety disorder, obsessive compulsive disorder, attention deficit hyperactivity disorder, conduct disorder, oppositional defiant disorder, enuresis, encopresis, anorexia nervosa, bulimia, transient tic disorder, Tourette's disorder, chronic motor or vocal tic disorder, alcohol abuse and dependence, substance abuse and dependence, post-traumatic stress disorder, and adjustment disorders), current use of sedative or psychotropic medication, current signs of or a history of fetal alcohol syndrome or exposure to heavy antenatal alcohol exposure, sensory impairment, history of traumatic brain injury with loss of consciousness exceeding 10 minutes, presence of diseases that may affect the CNS (e.g., meningitis, epilepsy), HIV [tested using the enzyme linked immunosorbent assay (ELISA)], less than 6 years of formal education, and lack of proficiency in English or Afrikaans. Prior to consent being obtained for participation in the study, a research social worker obtained collateral information from consenting parents, verifying the absence of medical, psychiatric and psychosocial problems. Participants in the two groups were matched for age (within 1 year), gender, language, socio-economic status and level of education (within 1 year). A total socio-economic status score was calculated by summing the category scores for family income (1-6), reversed employment category of participant's parent with the highest employment rank (Hollingshead reversed) (1-9), parent education (0-6), total assets (0-7), dwelling type (1-6) and bedroom cohabitation (1-7). During recruitment it was attempted to match the samples on smoking status, but this was not to be feasible since smoking was much more prevalent in the AUD participants. This positive association of smoking and alcohol use is well documented [35].

### Measures

#### Substance use

A revised version of the Timeline Follow-back procedure (TLFB) [36], a semi-structured, clinician-administered assessment of lifetime history of alcohol use and drinking patterns (i.e., frequency, quantity and density of alcohol consumption, including every phase from when participants first started drinking at least once per month to the present, including all periods of abstinence) was used in combination with the K-SADS-PL to elicit alcohol-use data. It was administered by a Psychiatrist on the day of screening. A standard drink was defined as one beer or wine cooler (340 ml), one glass of wine (150 ml) or a 45 ml shot of liquor. A similar procedure was carried out for each substance that the research participant acknowledged using.

#### Anthropometry

Weights and heights were measured according to standard anthropometric techniques [37] by a trained research assistant. Weight was measured using a digital weighing scale (Camry ED-301, China) and shoes and heavy outer layers of clothing were removed. Height was measured using a wall-mounted stadiometer without shoes, caps and hats. Indices investigated were height-for-age (stunting) and BMI (weight/height^2^)-for-age (underweight and overweight/obesity). For comparison of anthropometric indices to reference data in developing countries, *Z*-scores derived from the reference data were used [38]. The recently released World Health Organisation (WHO) growth reference for ages 5 to 19 years [20] were used as reference data. Height-for-age, expressed in *Z*-scores of the WHO growth reference was used to define stunting (< -2 standard deviations (SD), and BMI-for-age, expressed in *Z*-scores of the WHO growth reference were used to define underweight (*Z*-score < -2 SD) and overweight and obesity (> +1 SD) [20] using WHO AnthroPlus, Version 1.0.4 for Windows [39]. In order to compare the anthropometric data with findings from the most recent YRBS [4], weight-for-age (underweight: *Z*-score < -2 SD) and height-for-age (stunting: *Z*-score < -2SD) were also expressed in *Z*-scores of the sex-specific 2000 CDC reference curves growth reference data [40] using the Nutrition module of Epi Info (TM) 3.5.3 [41]. As in the 2008 YRBS, the International Obesity Task Force (IOTF) age-dependent BMI cut-off points for overweight and obesity (BMI ≥ 25 kg/m^2^) were used to determine prevalence of overweight/obesity [42] for comparison purposes.

#### Physical activity

A questionnaire, administered by the primary researcher, was used to estimate active and sedentary time. Physical activity was assessed as time in minutes per week using regular, weekly 'organised sporting activities' such as scheduled training and competitions for sports (e.g. athletics and rugby). Physical inactivity was measured as time in minutes per week using 'television watching' and 'computer use'. Information for each of the three domains was elicited using three responses: participation: yes or no; if yes, number of occasions per week and minutes per occasion. The total minutes per week was calculated for each domain. The domain for physical activity represented total weekly active time (minutes) and the two domains for physical inactivity were added to obtain total weekly sedentary time (minutes).

#### Energy intake

Energy intake (kilojoules) was estimated by three repeated 24-hour recalls, which has been shown to be appropriate for quantifying dietary intake in developing countries [43,44]. Furthermore, internal and external validity of this method has been found to be acceptable in adolescents aged ten years and older [45]. The 24-hour recall interviews were all conducted by the primary researcher, who was trained and standardised and knowledgeable on terminology and locally available food and beverages, on two non-consecutive week days and on one Monday to obtain Sunday intake. No data could be collected for Fridays and Saturdays as it was not feasible to conduct interviews on Saturdays and Sundays. Commonly used household measures and food pictures from the Dietary Assessment and Education Kit, developed by Steyn and Senekal [46], were used to assist with food portion size estimation. Estimated food portions were converted to grams using the the MRC Food Quantities Manual [47]. The energy consumption for each participant for each day was calculated using the South African Food Data System (SAFOODS) [48]. The average energy intake over the three 24-hour recall interviews was calculated to represent the observed intake distributions for energy (kilojoules). This energy intake variable did not include estimated energy from alcohol intake. Average daily alcohol energy intake was estimated from average daily alcohol intake (grams) per participant in the AUDs group as follows: The average daily alcohol intake (grams) was estimated from the alcohol-use data in the most recent phase of drinking as follows: 1) frequency of alcohol use (days per month) multiplied by average quantity of alcohol consumed (standard drinks per drinking day) to obtain average monthly standard drinks of alcohol consumed; 2) average monthly standard drinks of alcohol consumed was divided by 28 days to obtain average daily standard drinks of alcohol consumed; 3) average daily standard drinks of alcohol consumed was multiplied by 13.6 grams of alcohol per standard drink to obtain average daily alcohol intake in grams, which was converted to average daily alcohol energy (29 kilojoules per gram) to obtain average daily alcohol energy (kilojoules). Average daily alcohol energy was added daily energy intake from the observed intake distributions for each AUD participant to represent total estimated energy intake (kilojoules). Daily alcohol energy for the *n = 48 *light drinking control participants was not calculated as their alcohol life dose was negligible (mean 5.77 ± 12.46 standard drinks) and the contribution of alcohol energy to energy intake would therefore also be negligible.

### Procedures

The Committee for Human Research of Stellenbosch University approved all study procedures (N06/07/128). After eligibility was established, written consent from parents and written assent from participants was obtained. Participants were transported from their homes or schools to the testing site. After physical and psychiatric screening, the participants completed demographic self-report questionnaires. On the day of screening after inclusion in the sample, weight and height measurements were taken, physical activity data was collected and the first 24-hour recall interview was conducted. The remaining two 24-hour recalls were done on a Monday and one other week day thereafter. Participants were compensated for their time with gift vouchers. Confidentiality of all study information was maintained with the exception of statutory reporting requirements in newly-identified or ongoing threats to the safety of minor participants.

### Statistical analysis

All data were checked and cleaned before analysis. Descriptive statistics, including inspection of data for adherence to normal distributions, and group comparisons were computed using Stata/IC Version 11.1 for Windows [49]. Suitable transformations were applied to all variables with skewed distributions, as relevant. Mann Whitney U, chi-square and Fischer's exact tests were used to compare the socio-demographic and substance use variables between the Control and AUD groups. Statistical significance was defined at a level of p ≤ 0.05. Due to the paired nature of the data and possible confounding from the smoking imbalance between the Control and AUD groups, a multi-level mixed effects linear regression model was used to compare anthropometric indices of growth and weight status, physical activity and energy intake between the two groups. A pairing variable was created according to the matched pairs in the sample and served as the level variable in the model. Transformed outcome variables were used where relevant. Comparisons were done with adjustment for gender, smoking status, physical activity (total weekly active time) and total estimated energy intake (including alcohol energy). Categorical physical activity variables (participate yes/no) were compared using the Chi-square or Fischer's exact tests. Prevalence of stunting, underweight and overweight/obesity using the WHO growth references were compared between the Control and AUD groups using the Chi-square and Fischer's exact tests.

After confirming that assumptions for regression analyses were met, relationships between anthropometric categories and AUD group membership were investigated for the total sample and within genders, using multi-level mixed effects logistic regression. Outcome variables were WHO anthropometric *Z*-score categories for stunting, underweight and overweight/obesity [20], and group status, gender, smoking status, physical activity and total estimated energy intake (including alcohol energy) were entered as independent variables. The pairing variable served as the level variable.

## Results

### Demographic and substance use characteristics

A total of 184 adolescents were recruited and screened, of whom 22 were excluded as screen failures due to a range of exclusion criteria, including cannabis and methamphetamine use, and DSM-IV Axis I diagnoses, resulting in a final sample of 162. The Control and AUD groups were successfully matched for age, education level, gender, language, ethnicity and socio-economic status (Table 1). As expected, AUD adolescents had significantly greater alcohol exposure than controls (Table 1). There was no significant difference in age of onset of drinking and participants who had ever used alcohol started drinking around 12 years of age. Almost all (95%, *n = 77*) adolescents in the AUDs group had a 'weekends-only' style of alcohol consumption. The regular drinking frequency (days per month) and regular drinking quantity (standard drinks per month) indicates an average consumption of about 13 drinks per drinking day, which is indicative of a binge drinking pattern. A greater proportion of participants in the AUDs group smoked compared to the Control group, and lifetime tobacco dose (total number of cigarettes smoked in lifetime) was greater in the AUDs group; a significantly greater percentage of participants in the AUDs group had also experimented with cannabis. The results on cannabis and methamphetamine indicate very low use in both groups, with no other drug use, although cannabis use was significantly greater in the AUDs group compared to the Control group (Table 1).

**Table 1:** Confirmatory Analyses of Socio-demographic and Alcohol Grouping Measures and Substance use characteristics of the sample (*n = 162*)

	Control Group (*n = 81*)	AUDs Group (*n = 81*)		
	***M *(*SD*) or %**	***M *(*SD*) or %**	**U/χ**^**2**^	**p-value**

**Socio-demographics**				
Age	14.76 (0.78)	14.92 (0.74)	-1.19	0.235
Education level ^a^	7.79 (0.85)	7.85 (0.74)	-0.43	0.666
%Male	42	42	0.00	1.000
%Female	58	58		
% Afrikaans-speaking	69	69	0.00	1.000
%English-speaking	31	31		
% Mixed Ancestry	97.6	97.6		0.497
% White	1.2	0		
% Black	1.2	0		
Total Socio-economic status score ^b^	28.19 (5.80)	24.85 (5.93)	1.34	0.179

**Alcohol Use**				
% Never consumed alcohol	41	0		
% Never intoxicated	93	0		
%Light drinker ^c^	59	0		
% Alcohol abuse ^d^		2.5		
% Alcohol dependence ^e^		97.5		
% Weekends-only drinking style in most recent drinking phase ^f^		95		
Drinking onset age (years) in participants that have drunk alcohol	12.25 (1.66)	12.04 (1.70)	0.57	0.567
Alcohol lifetime dose ^g^	5.77 (12.46)	1493.69 (1511.53)	-11.04	**< 0.001**
Age of first intoxication		12.83 (1.15)		
Age of onset of regular drinking		12.91 (1.11)		
Regular drinking duration (months)		23.78 (15.91)		
Regular drinking frequency (days/month) in most recent drinking phase		5.01 (2.87)		
Regular drinking quantity per month ^h^		65.78 (57.96)		

**Tobacco Use**				
% Never smoked tobacco	59	17		**< 0.001**
% Light smokers (lifetime < 100 cigarettes)	35	31		
% Regular smokers (lifetime > 100 cigarettes)	6	52		
Smoking onset age (years) in light smokers	12.53 (1.62)	12.44 (1.96)	-0.19	0.846
Smoking onset age (years) in regular smokers	13 (0.71)	12.36 (1.46)	0.96	0.339
Lifetime tobacco dose of all smokers ^i ^	86.42 (442.80)	1417.59 (2762.60)	-7.02	**< 0.001**

**Other Substance Use**				
% Never used cannabis	89	42	34.35	**< 0.001**
Lifetime cannabis dose ^j ^	0.12 (0.37)	4.08 (7.40)	-6.33	**< 0.001**
% Never used methamphetamine	100	96		0.245
Lifetime methamphetamine dose ^k^	0	1.0 (0.56)		
% Never used any other drugs	100	100		

*Notes*: For all variables not presented as percentages, means are presented with standard deviations in parentheses. Continuous variables compared using the Mann Whitney U Test and categorical variables compared using the Chi-Square or Fisher's exact test.

^a ^Years of successfully completed education

^b ^Total Socio-economic status score: Sum of Family income (1-6), Reversed employment category of participant's parent with the highest employment rank (Hollingshead reversed) (1-9), Parent education (0-6), Total assets (0-7), Dwelling type (1-6) and Bedroom cohabitation (1-7) - Maximum = 41

^c ^Less than 100 standard drinks of alcohol consumed in lifetime

^d ^Greater than 100 standard drinks of alcohol consumed in lifetime with a DSM-IV diagnosis of alcohol abuse

^e ^Greater than 100 standard drinks of alcohol consumed in lifetime with a DSM-IV diagnosis of alcohol dependence

^f ^Style of drinking followed in the most recent phase of drinking

^g ^Total number of standard drinks of alcohol consumed in lifetime

^h ^Average standard drinks of alcohol consumed per month

^i ^Total number of cigarettes smoked in lifetime

^j ^Total number of 'joints' smoked in lifetime;

^k ^Total number of 'straws' (hits) of methamphetamine in lifetime

### Anthropometry, Physical Activity and Energy Intake

Complete anthropometric data sets were collected for 157 participants (Control group: *n = 79*, AUDs group: *n = 78*) and complete physical activity and energy intake data sets were collected for 160 participants (*n = 80 *per group). Heights and weights could not be obtained for 5 participants in the sample (Control group: *n = 2*, AUDs group: *n = 3*) and dietary intake and physical activity data could not be obtained for 2 participants (*n = 1 *per group). Weight, height, height-for-age and BMI-for-age, expressed in *Z*-scores of the WHO growth reference for ages 5 to 19 years [20], did not differ significantly between groups and there were no group by gender differences in height-for-age and BMI-for-age (Table 2). Females in the sample had a significantly greater BMI-for-age (z = 2.34; p = 0.019) compared to males when adjusting for group status, smoking status, physical activity and total estimated energy intake (including alcohol). A significant positive association was found between height-for-age and total estimated energy intake (z = 2.72; p = 0.007) when adjusting for group status, gender, smoking status and physical activity. No significant relationship was found between BMI-for-age and total estimated energy intake (z = 1.50; p = 0.132). Most participants in both groups had a height-for-age within the normal WHO reference range, with one male AUD participant having a height-for-age greater than +2SD. Body mass index- for-age was within the WHO normal reference range for approximately two thirds of participants in both groups. The proportion of stunted, thin and overweight/obese participants did not differ significantly between groups, and there was no gender by group interactions (Table 3). In both groups, more than double the numbers of females than males were overweight/obese (Table 3).

**Table 2:** Comparison of anthropometry, physical activity and energy intake between Control and AUD groups

	Control Group		AUDs Group			
		**%**		**%**	**p-value**	**z**

**Anthropometry**						
Weight (kg)	52 (46-62)		53 (46-62)		0.745	-0.32
Height (m)	1.60 (1.56-1.65)		1.60 (1.53-1.65)		0.487	0.70
Height-for-age *Z*-score	-0.58 (1.03)		-0.59 (1.01)		0.672	0.42
Males	-0.73 (1.07)		-0.45 (1.14)		0.348	0.94
Females	-0.47 (0.99)		-0.69 (0.89)		0.673	-0.42
Body mass index-for-age *Z*-score	0.29 (1.42)		0.25 (1.38)		0.821	-0.23
Males	0.12 (1.52)		-0.11 (1.42)		0.219	-1.23
Females	0.41 (1.34)		0.52 (1.31)		0.327	0.98
						
**Physical activity and inactivity**						
Participate in physical activities (%yes) *		33		33		
Males (%yes) *		48		39		
Females (%yes) *		21		28		
Total weekly active time (minutes) ^a^	0 (0-120)		0 (0-120)		0.740	-0.33
Males: weekly active time	0 (0-240)		0 (0-240)		0.382	-0.87
Females: weekly active time	0 (0-0)		0 (0-60)		0.690	0.40
Participate in sedentary activities (%yes) *		100		99		
Total weekly sedentary time (minutes) ^b^	1001 (570)		1091 (569)		0.949	0.06
Males: weekly sedentary time	937 (590)		1093 (561)		0.349	0.94
Females: weekly sedentary time	1047 (558)		1089 (580)		0.327	-0.98
						
**Energy intake**						
Energy (kilojoules), excluding alcohol energy ^c^	8965 (7240-10661)		10063 (8245-11683)		**0.047**	1.98
Males: energy excluding alcohol	9461 (8010-10835)		11206 (9622-12496)		**0.034**	2.11
Females: energy excluding alcohol	8342 (7003-9944)		9291 (7923-10941)		0.254	1.14
Total estimated energy (kilojoules), including alcohol energy ^d^	8965 (7240-10661)		11028 (9072-13014)		**< 0.001**	4.20
Males: energy including alcohol	9461 (8010-10835)		11684 (10181-13521)		**< 0.001**	3.69
Females: energy including alcohol	8342 (7003-9944)		10481 (8847-11726)		**0.005**	2.81

*Notes: *Variables are median and interquartile range except for height-for-age *Z*-score, body mass index-for-age *Z*-score, total weekly sedentary time, which are mean and standard deviation

n-values: Anthropometry: Control *n = 79*; AUDs *n = 79*; Physical activity and inactivity and Energy intake: *n = 80 *per group; males *n = 33 *and females *n = 47*

Height-for-age and body mass index-for-age expressed in *Z*-scores of the World Health Organisation Growth Reference for ages 5 to 19 years [20]

Smoking group includes light smokers (lifetime < 100 cigarettes) and regular smokers (lifetime > 100 cigarettes)

Multilevel mixed-effects linear regression used to compare groups, adjusting for gender, smoking status, physical activity (total weekly active time) and total estimated energy intake (including alcohol energy)

^a ^Total weekly active time: sum of minutes per week spent doing regular (weekly) organised sporting activities

^b ^Total weekly sedentary time: sum in minutes per week spent watching television and using a computer

^c ^Energy intake, excluding estimated average daily alcohol energy

^d ^Total estimated energy intake, including average daily alcohol energy estimated from average daily alcohol intake (grams) per participant in the alcohol use disorder group

* No significant differences between Control and AUD groups using χ^2 ^or Fischer's exact test

**Table 3:** Comparison of height-for-age and body mass index-for-age according to WHO Growth References (5-19 years) *Z*-score cut-offs in Control (*n = 79*) and AUD (*n = 78*) groups and by gender between groups

	Groups*		Males*		Females*	
	%Control *n*	%AUDs *n*	%Control *n*	%AUDs *n*	%Control *n*	%AUDs *n*
**Height-for-Age**						
Stunting < -2SD	10.1 *8*	6.4 *5*	11.8 *4*	9.1 *3*	8.9 *4*	4.4 *2*

**Body mass index-for-Age**						
Underweight < -2SD	5.1 *4*	2.6 *2*	8.8 *3*	3.0 *1*	2.2 *1*	2.2 *1*
Overweight and Obese > +1SD	25.3 *20*	29.5 *23*	17.7 *6*	18.2 *6*	31.1 *14*	37.8 *17*

*Notes: *Body mass index (kg/m2), calculated by dividing weight (kilograms) by height (meters) squared

n-values: Control group: *n = 34 *males and *n = 45 *females; AUDs Group: *n = 33 *males and *n = 45 *females

* No significant differences between Control and AUD groups or by gender between groups using χ^2 ^or Fischer's exact tests

No significant differences in the number of participants engaging in organised sports (active time) were found in the total sample, nor was there a group by gender interaction (Table 2). There was also no significant difference in the total weekly active time between groups or by gender (Table 2). All participants in the sample, excepting one in the AUDs group, participated in sedentary time activities, with no differences in total weekly sedentary time between groups nor any group by gender interactions (Table 2).

Daily energy intake, excluding alcohol energy, was significantly higher in the AUD adolescents compared to the control adolescents for the entire group, and for males, but not for females. However, when estimated alcohol energy was added, energy intake in the AUD participants was significantly higher for both males and females (Table 2)

Mixed effects logistic regression showed an increased odds ratio (OR 1.26) of AUD participants being overweight/obese when compared to control participants, after adjusting for gender, smoking status, physical activity (total weekly active time) and total estimated energy intake. Within the total sample, females had an increased odds ratio (OR 2.60) of being overweight/obese compared to males with adjustment for group status, smoking, physical activity and total estimated energy intake. Females with AUDs had a greater odds ratio (OR 2.42) compared to males of being overweight/obese, adjusting for smoking status, physical activity and total estimated energy intake, while no increased odds ratio was found for males with AUDs (OR 0.76) (Table 4).

**Table 4:** Multilevel mixed-effects logistic regression with overweight/obesity

	Odds Ratio	p-value	95% CI	
**Overweight/Obesity: Body mass index-for-age *Z*-score > +1SD**				
Control Group	1.00			
AUDs Group	1.26	0.647	0.46	3.41
Males	1.00			
Females	2.61	0.053	0.99	6.87
Non-smoking	1.00			
Smoking	0.69	0.498	0.23	2.03
Total weekly active time	1.00	0.387	1.00	1.00
Total estimated energy intake (including alcohol energy)	1.00			

**MALES: Overweight/Obesity: Body mass index-for-age *Z*-score > +1SD**				
Control Group	1.00			
AUDs Group	0.75	0.720	0.16	3.48
Non-smoking	1.00			
Smoking	1.04	0.967	0.17	6.31
Total weekly active time	1.00	0.345	0.99	1.00
Total estimated energy intake (including alcohol energy)	1.00	0.075	1.00	1.00

**FEMALES: Overweight/Obesity: Body mass index-for-age *Z*-score > +1SD**				
Control Group	1.00			
AUDs Group	2.42	0.190	0.65	9.02
Non-smoking	1.00			
Smoking	0.43	0.248	0.10	1.81
Total weekly active time	1.00	0.685	1.00	1.00
Total estimated energy intake (including alcohol energy)	1.00	0.025	1.00	1.00

*Notes: *Smoking group includes light smokers (lifetime < 100 cigarettes) and regular smokers (lifetime > 100 cigarettes)

Control group *n = 79 *(males *n = 34 *and females *n = 45*); AUDs group *n = 78 *(males *n = 33 *and females: *n = 45*)

## Discussion

The results of this study show that the growth and weight status of a group of healthy, treatment-naive adolescents with "pure" AUDs (without comorbid substance use disorders or comorbid psychiatric, including externalising, disorders) is comparable to a matched group of light/non-drinking control adolescents and is in line with the growth and weight status of the South African adolescent population. There were a greater proportion of overweight/obese females in both groups compared to males, with this percentage being slightly greater, although not significantly so, in the AUDs group. Our analyses suggest that within this developing country setting, adolescent females with AUDs have an increased risk of being overweight/obese compared to adolescent females without AUDs, adjusting for smoking, physical activity and estimated energy intake (including alcohol energy).

When using the recommended WHO growth reference for ages 5 to 19 years, weight, height, height-for-age *Z*-scores and BMI-for-age *Z*-scores did not differ significantly between the Control and AUD groups, and a similar prevalence of stunting, underweight and overweight and obesity were found in the groups. These anthropometric findings were compared to the findings of the 2008 YRBS [4], in order to contextualise the findings within the South African adolescent population (Table 5). Stunting and underweight in both groups in our sample were lower than the YRBS prevalence nationally and in the mixed ancestry population, but similar to the stunting prevalence in the Western Cape Province. In the AUDs group, overweight and obesity prevalence was greater than in the YRBS national, provincial and Mixed Ancestry population prevalence, while Control group prevalence was similar to the YRBS proportions [4]. With the same anthropometric references used in the YRBS, more overweight/obesity in the AUDs group compared to the controls was evident, with higher prevalence in females in both groups, particularly within the AUDs group (Table 5). All indices point to the fact that prevalence of overnutrition exceeded undernutrition in this sample. Female AUD participants showed an increased risk for overweight/obesity (OR = 2.42). However, studies in larger samples are needed to confirm these findings.

**Table 5:** Comparison of prevalence of underweight and stunting in Control (*n = 79*) and AUDs (*n = 78*) groups with 2008 Youth Risk Behaviour Survey findings in the Western Cape Province (WC) (*n = 1159*), nationally (*n = 9862*) and for mixed ancestry population (*n = 1423*)

	Groups		YRBS		
	**% Control** **% Males** **%Females**	**%AUDs** **%Males** **%Females**	**%WC** **%Males** **%Females**	**%National** **%Males** **%Females**	**%Mixed** **Ancestry**

**Stunting: Height-for-age *Z*-score < -2SD**	**8.9** 8.8 8.9	**10.3** 9.1 11.1	**9.7** 9.8 9.6	**13.1** 15.2 11.1	**13.6**

**Underweight: Weight-for-age *Z*-score < -2SD**	**7.6** 11.8 4.4	**6.4** 9.1 4.4	**6.5** 8.0 5.2	**8.4** 12.0 4.9	**9.4**

**Overweight and obese: Body mass index ≥ 25 kg/m**	**22.8** 17.7 26.7	**29.5** 18.2 37.8	**25.5** 13.7 36.3	**25.0** 14.5 35.0	**22.4**

Notes: Height-for-age and Weight-for-age according to gender-specific 2000 CDC reference [40]*Z*-score cut-offs (Epi Info (TM) 3.5.3) used in 2008 Youth Risk Behaviour Survey [4]

Body mass index according to International Obesity Task Force cut-offs for body mass index for overweight and obesity by gender for ages 2 to 18 years [42] used in 2008 Youth Risk Behaviour Survey [4]

AUDs: alcohol use disorders

Participation in physical activity was low in both groups with only a third of adolescents participating in any organised sporting activities. Average weekly active time, represented by organised regular sporting activities, was not significantly different between groups (Control group: median = 0; mean = 83 minutes; AUDs group: median = 0, mean = 104 minutes). Physical activity in both groups was well below the WHO global recommendation for physical activity (five to 17 year olds) of 60 minutes of moderate to vigorous physical activity daily [50]. It is possible that informal recreational activity such as dancing and playing sport with friends may contribute to activity levels in adolescents who are recreationally active on a regular basis. However, these low physical activity levels are in line with YRBS findings that show widespread inadequate levels of physical activity, especially in the Western Cape and among females [4,10]. This inactivity may contribute to positive energy balance and the observed overweight/obesity prevalence.

Based on the predominance of sedentary behaviour in the total sample, the daily estimated energy requirement (EER) for sedentary adolescents (aged 15 years) was used for interpretation of energy intake data (males: 9337 kilojoules per day, females: 7270 kilojoules per day; and weighted average EER for males and females for total sample: 8138 kilojoules per day) [51]. Total estimated energy intake (including alcohol) for the AUDs group was considerably greater than the EER for sedentary adolescents (weighted average EER as mentioned above), whereas that of the Control group was only slightly greater than the weighted average EER. Total estimated energy intake in females in the Control group and in both genders in the AUDs group exceeded sedentary EERs for females and males aged 15 years. As expected, total estimated energy intake (including alcohol) was significantly greater in the AUDs group compared to the Control group, since energy intake before the addition of alcohol energy was significantly higher in the AUDs group compared to the controls. This suggests that when compared to adolescents without AUDs, adolescents with AUDs may have higher intakes of energy, possibly due to energy-dense food choices, which is then further increased by added alcohol energy. It therefore seems reasonable to suggest that heavy drinking adolescents are ingesting alcohol energy in addition to usual food and beverage energy, resulting in diets high in total energy. Previous research showed that adolescents with AUDs were less likely to eat a balanced diet [52] and that adolescent alcohol and tobacco use is associated with consumption of energy-dense snacks and beverages [27,30]. A recent study reported that earlier alcohol, tobacco and other drug use, depression, increased fighting and reduced optimism may lead to unhealthy increases in weight [53]. The inherent limitations of recall data for alcohol use, energy intake and physical activity are applicable to this study and the results therefore need to be interpreted with caution.

The higher frequency and risk of overweight/obesity in female adolescents as a whole, while being more pronounced in the AUD females, is particularly relevant in view of obesity development being linked to four critical periods, namely intrauterine life, infancy, the period of adiposity rebound (ages 5 to 7 y), and adolescence [54] and since the transition from adolescence to early adulthood is a period characterised by considerable increase in obesity incidence [55]. These findings become more pertinent in view of the substantial evidence that risk behaviours, such as alcohol use [56] and weight status [57] show a strong degree of tracking from adolescence into adulthood. Longitudinal data also shows that low physical activity in adolescence tracks into adulthood [58]. In addition, the odds of overweight/obese adolescents reporting a diagnosis for two cardiovascular risk factors by young adulthood is 1.5 to 2 times higher than for normal-weight adolescents, regardless of BMI in young adulthood [59]. Furthermore, physical inactivity [60] and smoking [61] have been shown to predict future obesity, in particular abdominal ("high risk") obesity by early adulthood. These adolescent behaviour patterns are therefore not transient in nature and are likely to have important impacts on long-term behaviour patterns and health outcomes. Overweight/obesity results in a considerable burden of death, premature death and disability in adult South African's [62], and is very much in line with the growing pandemic worldwide in developed as well as developing countries. This trend seems unlikely to be reversed as developing countries continue to struggle with the widespread availability of nutritionally poor, but cheap foods.

## Conclusion

In this cross-sectional comparison, anthropometric indices of growth and weight status of participants in the Control and AUD groups were generally comparable. However, there is an indication that adolescent females with AUDs may have an increased risk for being overweight/obese compared to similar adolescent females without these disorders within this developing setting. Moreover, the presence of an AUD in this adolescent sample was associated with higher energy intake, likely due to greater intakes of energy-dense food choices, exacerbated by added alcohol energy. This result is consistent with the literature suggesting that heavy alcohol use is associated with unhealthy increases in weight. The persistence of these patterns has the potential to initiate early development of nutrition-related chronic diseases and burdens in adulthood. These findings need further exploration in longitudinal, well-controlled studies, in order to elucidate the impacts of heavy alcohol use on energy balance, growth and weight status in adolescents as they age. This assessment of growth and weight status in adolescents with AUDs contributes to the understanding of possible impacts of heavy alcohol consumption on important aspects of adolescent development.

## List of abbreviations

AUDs: Alcohol Use Disorders; BMI: Body mass index; DSM-IV: American Psychiatric Association's 4th Diagnostic and Statistical Manual; ELISA: Enzyme linked immunosorbent assay; EER: Estimated Energy Requirement; K-SADS-PL: Schedule for Affective Disorders and Schizophrenia for School Aged Children (6-18 Years) Lifetime Version; MRC: Medical Research Council; TLFB: Timeline Follow-back procedure; SAFOODS: South African Food Data System; SSAGA-II: Semi-Structured Assessment for the Genetics of Alcohol (SSAGA-II); SUDs: Substance Use Disorders; WHO: World Health Organization; YRBS: Youth Risk Behaviour Survey

## Competing interests

The authors declare that they have no competing interests.

## Authors' contributions

CEN, MS, PDC, GF were responsible for the study concept and design. CEN, PDC contributed to the acquisition of the data. RL, CEN, MS, GF assisted with data analysis and interpretation of findings. CEN drafted the manuscript. MS, RL, PDC, GF provided critical revision of the manuscript. All authors read and approved the final manuscript.
